# Rural-urban disparities in Oral Health-related Quality of Life for middle-aged and older adults with diabetes in Taiwan

**DOI:** 10.3389/fpubh.2023.1162201

**Published:** 2023-04-25

**Authors:** Hsueh-Fen Chen, Yun-Ti Lin, Jia-Ying Lin, Huey-Er Lee

**Affiliations:** ^1^Department of Healthcare Administration and Medical Informatics, College of Health Sciences, Kaohsiung Medical University, Kaohsiung City, Taiwan; ^2^Department of Medical Research, Kaohsiung Medical University Hospital, Kaohsiung City, Taiwan; ^3^Center for Big Data Research, Kaohsiung Medical University, Kaohsiung City, Taiwan; ^4^Management of Planning and Coordinating Center, Yuan's General Hospital, Kaohsiung City, Taiwan; ^5^Department of Dentistry, Yuan's General Hospital, Kaohsiung City, Taiwan

**Keywords:** Oral Health-related Quality of Life, social determinants, diabetes, disparities, diabetes-related complications, Oral Health Impact Profile

## Abstract

**Background:**

Public health faces a significant challenge in reducing rural–urban disparities in diabetes. Since dietary control is part of the medical regimen for diabetes management, how diabetic patients perceive the impact of oral health on their quality of life is critical. The present study aimed to compare the Oral Health-related Quality of Life (OHRQoL) between rural and urban diabetic patients.

**Methods:**

The study design was cross-sectional. The study sample included 831 self-reported diabetic patients, extracted from the first wave of the new-cohort Taiwan Longitudinal Study on Aging survey (NC_TLSA) that comprised a nationally representative sample of community-dwelling adults aged 50 and above in Taiwan. The composite score generated from the Oral Health Impact Profile-7 (OHIP-7), which has seven questions, was used to construct two OHRQoL measures, the severity of perceived poor OHRQoL and the prevalence of poor OHRQoL. These two OHRQoL measures were treated as dichotomous variables. Multivariate logistic regression models were applied for analysis.

**Results:**

Rural diabetic patients had a higher likelihood of experiencing the severity of perceived poor OHRQoL than those in urban areas (OR = 2.40, 95% CI: 1.30–4.40). Although rural diabetic patients also had a higher prevalence of poor OHRQoL than urban diabetic patients, the difference was not significant (OR = 1.47, 95% CI: 0.95–2.28). Social determinants, such as education, are essential factors attributed to both OHRQoL measures.

**Conclusion:**

Overall, rural diabetes community-dwelling patients had a poorer OHRQoL than those in urban areas. Given a bidirectional relationship between oral health and diabetes, improving oral health in rural areas may be a critical avenue to improve the quality of diabetes care in rural areas.

## Introduction

Oral health is critical to overall health. Based on Locker's conceptual framework, poor oral health leads to physical and psychosocial health problems (1). For example, with poor oral health conditions, individuals experience pain, cannot enjoy food due to chewing difficulty, and lose interest in socialization and networking due to poor pronunciation or bad breath. Following Locker's concept, Slade and Spencer developed the Oral Health Impact Profile (OHIP) to measure Oral Health-related Quality of Life (OHRQoL), which evaluates how patients perceive the impact of oral health on their quality of life in seven domains: functional limitation, physical pain, psychological discomfort, physical disability, psychological disability, social disability, and handicap (2). Frequent visits to the emergency department due to poor oral health highlights the impact of oral health on overall health and wellbeing (3).

For diabetic patients, oral health is even more crucial. Dietary control is part of the medical regimen to stabilize blood sugar and reduce the likelihood of diabetes-related complications (e.g., blindness due to retinopathy). Poor oral health would limit food choices and increase the difficulty for diabetic patients in managing their daily life. Most importantly, there is a causal relationship that runs both ways between diabetes control and oral health conditions (4). Diabetic patients are more likely to develop different forms of oral health problems, such as periodontal disease, dry mouth, and dental caries, and to develop few remaining natural teeth than those without diabetes (5, 6). Diabetic patients with poor oral health also have poorer diabetes control due to insulin resistance than those without poor oral health (7, 8), which increases the likelihood of having diabetes-related complications. As the prevalence of diabetes among individuals aged 20–79 years is expected to increase from 9.7% in 2021 to 12.6% in 2045 (9), oral health for diabetes patients is regarded as a critical issue from the perspective of public health.

Although several studies investigated clinical dental problems for diabetic patients, only a few focused on self-reported quality of life. Some studies measured general health quality of life, such as the number of physically and mentally unhealthy days (10). Others used OHIP to measure OHRQoL. Previous studies examined the impact of periodontal diseases on the OHRQoL between patients with and without diabetes and found mixed findings (11–13). Others identified risk factors associated with the poor OHRQoL of diabetic patients and found several risk factors, including, but not limited to, dry mouth sensation, the use of a removable prosthesis, untreated dental caries, periodontal disease, unmet denture needs, low income, and poor oral hygiene (14, 15). Based on the national data with a sample of 2,945 community dwellers in the United States, the study found that diabetic patients were more likely to experience poorer OHRQoL than those without diabetes (15).

There is a rural–urban discrepancy in diabetes incidents and diabetes-related complications. Compared to the urban population, rural individuals are at a higher risk of having diabetes, receive poorer process of diabetes care (e.g., high blood pressure and hemoglobin A1c), and have poorer outcomes (e.g., nephropathy, low-extremity amputation, and mortality) (16–20). In addition, there is a rural–urban disparity regarding oral health conditions. Rural populations generally have higher periodontal disease and tooth decay rates, with fewer remaining natural teeth and receiving less preventive dental care than urban populations (21, 22). A study based on the general population in Quebec, Canada found that rural community dwellers had poorer OHRQoL than those who resided in urban areas (23). Given the discussion above, one would expect a rural–urban discrepancy regarding the OHRQoL among diabetic patients. However, to the best of our knowledge, evidence regarding discrepancies in OHRQoL of diabetic patients due to rurality is lacking. Diabetes and oral health are the top priorities for improving rural population health (24, 25). Evidence regarding OHRQoL for diabetic patients in rural areas would help policymakers find strategies to improve rural population health.

The present study aimed to compare the difference in OHRQoL between rural and urban diabetic patients by using the national data of Taiwan. Approximately 20% of the Taiwanese population reside in rural areas (26), similar to some developed countries, such as the United States (27). In 1995, Taiwan implemented a single-payer universal health insurance program that covered 99% of the Taiwanese population with low-cost healthcare (28). However, nearly 30 years later, rural–urban disparities in diabetes and oral health care remain (18, 19, 29). Empirical evidence from the present study fills the existing literature gap and provides direction regarding how to deliver better care to people in rural communities.

## Materials and methods

### Data source

The primary data source of the present study is the first wave of the new-cohort Taiwan Longitudinal Study on Aging survey (NC_TLSA), which the Taiwan Health Promotion Administration, Ministry of Health and Welfare launched in 2015. The NC_TLSA comprised 5,304 individuals, a nationally representative sample of community-dwelling adults aged 50 and above, with a response rate of 70.7% (30). The survey questions include six dimensions: (a) personal information, marriage status, and residence history; (b) household structure, satisfaction with a living arrangement, and interaction with children, relatives, and others; (c) health status, health utilization, and hygiene behaviors; (d) social support and exchange; (e) work history; and (f) social participation and physical safety (30). The data were collected through face-to-face interviews. TLSA provides variables necessary for the present study, such as the Oral Health Impact Profile (OHIP) commonly used to assess OHRQoL, the diseases that individuals had, and the locations where individuals lived at the time of the interview. The sampling process, survey questions, and data validity of the NC_TLSA are available on the TLSA website (30).

### Study design and study sample

**Figure 1:**
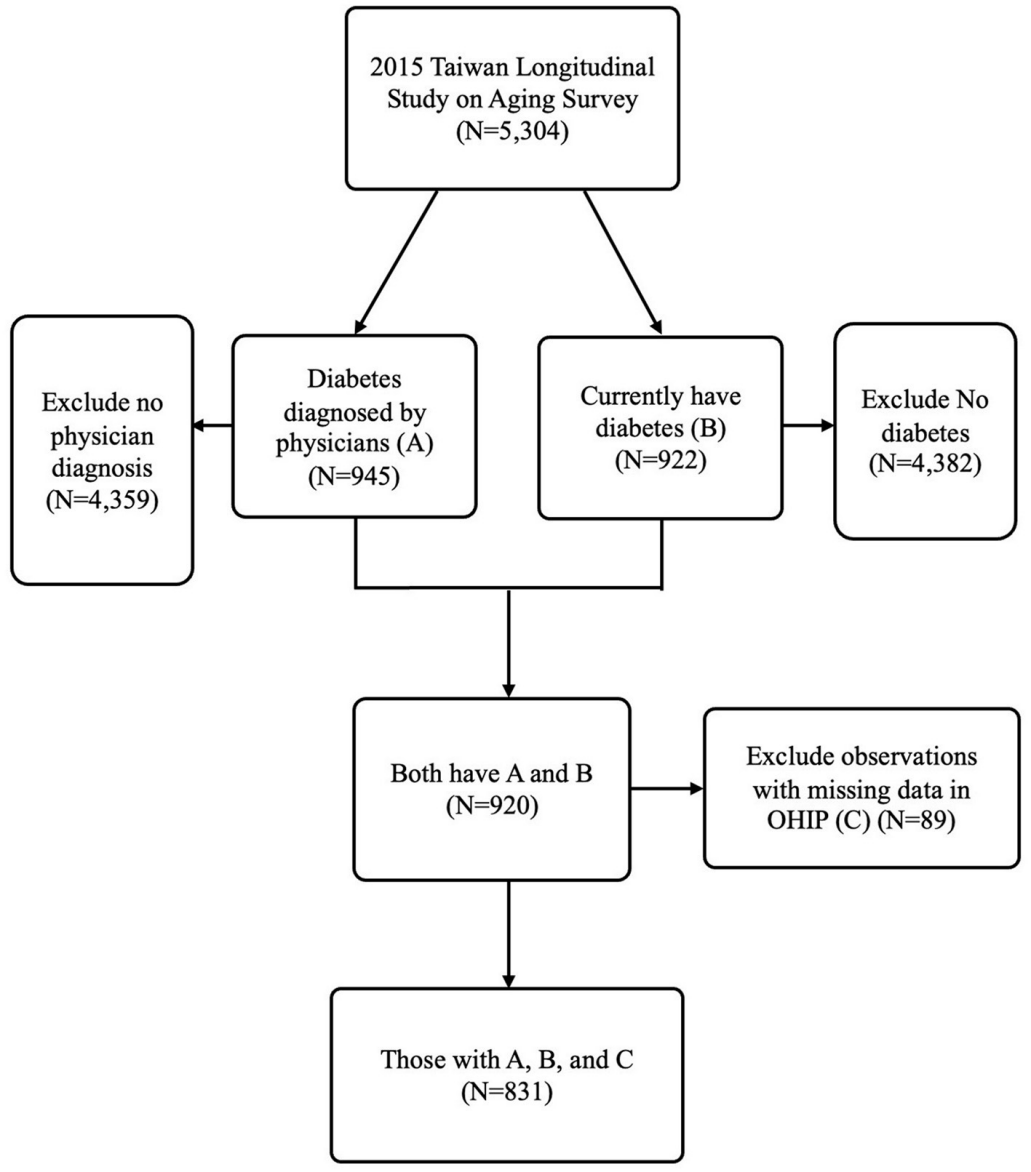
Study sample flow chart.

The study was cross-sectional, with community-dwelling adults aged 50 and older who self-reported having diabetes as the study sample. The TLSA had two questions. One is, “Have you ever been told by a doctor that you have diabetes?” The other is, “Do you still have the disease at the time of the interview?” Individuals who answered “yes” to both survey questions were defined as having self-reported diabetes in the present study. In total, 920 individuals answered “yes” to both questions. The present study further excluded 89 individuals with missing OHIP data. The final qualified study sample was 831 community-dwelling diabetic patients in the present study. Figure 1 presents the selection process for the study sample.

### Variable measures

#### Dependent variables

OHRQoL, the primary outcome variable of interest, was assessed by the OHIP-7. The original OHIP has 49 survey questions (OHIP-49). Later, the number of questions from the OHIP-49 was shortened into different versions (e.g., OHIP-14 with 14 questions and OHIP-7 with seven questions) and used in other countries.

OHIP-7—a validated Mandarin version of OHIP in NC_TLSA (31), which had been used in a previous study (32, 33)—surveys individuals' experiences related to teeth or denture problems in the past 12 months at the time of the interview through seven questions: (1) “Have you ever been aware of teeth or dentures problems?”, (2) “Have you ever been interrupted in a meal because of teeth or dentures problems?”, (3) “Have you ever experienced discomfort while eating because of teeth or dentures problems?”, (4) “Have you ever had difficulties with concentration because of teeth or dentures problems?”, (5) “Have you ever experienced difficulties with pronunciation because of teeth or dentures problems?”, (6) “Have you ever confronted difficulties with performing daily life because of teeth or dentures problems?” and (7) “Have you sensed taste deterioration because of teeth or dentures problems?”. Each survey question was rated on a 5-point Likert scale (never = 0, rarely = 1, occasionally = 2, often = 3, and very often = 4). The Likert scale from the seven survey questions was used to construct a composite score to assess how frequently individuals experienced poor OHRQoL.

Following previous studies (15, 23), the composite score generated from OHIP-7 was used to construct two OHRQoL measures—the severity of perceived poor OHRQoL and the prevalence of poor OHRQoL. The severity of perceived poor OHRQoL was based on a summative score, ranging from 0 to 28, generated from seven questions in OHIP-7. A higher score means a poorer OHRQoL. The summative score was then categorized as a dichotomous variable using the 85th percentile as a cutoff point (a score ≥8 was coded as one and zero otherwise), which was commonly used in the previous study (15). The prevalence of poor OHRQoL is the percentage of individuals rated “often” or “very often” for one or more questions in OHIP-7. The prevalence of poor OHRQoL was also dichotomized. Individuals who rated “often” or “very often” for at least one question in OHIP-7 were coded as one and zero otherwise (15, 23).

#### Key independent variable

The key independent variable of interest is the place of residence, categorized as rural and urban (urban as the reference group). TLSA provides five categories of residential areas: metropolitan, any major city at the province and county levels, towns, and rural areas. Individuals who lived in rural areas were coded as one and zero for those in urban areas as the reference group.

#### Control variables

The present study chose covariates based on the Andersen Behavioral Model of Health Care Utilization, focusing on the association between health outcomes and individuals' predisposing, enabling, and health need factors (34). Predisposing factors included a series of dummy variables, including age (50–64 and 65+ years), sex (men and women), and marital status (married and others, such as single or divorced). Enabling factors included dichotomous variables: employment status (with or without a job), education (with or without a high school diploma), living status (living alone or not), and family/social support (satisfaction or no satisfaction with family or social support). Finally, health need factors included two dummy variables: (1) functional condition (with or without at least one difficulty in the activity of daily living (ADL) or instrument activity of daily living (IADL)) and (2) poor health behaviors (with or without having behaviors related to drinking, smoking, or chewing betel), as well as a count variable for the number of comorbidities.

#### Analytical approach

The present study applied the bivariate analyses by using a *t*-test and the chi-squared test to compare the differences in study variables between rural and urban community-dwelling diabetes patients. Because the severity of poor perceived OHRQoL and the prevalence of poor OHRQoL were dichotomous, the present study applied multivariate logistic regression models to test the hypotheses. The odds ratio and 95% confidence intervals were reported with the significant level defined as a *P*-value of < 0.05. The odds ratio higher than one means a higher likelihood of experiencing poor OHRQoL. The SAS version 9.4 was applied for the analyses.

## Results

Figure 1 shows the study sample selection process from national survey data based on the middle-aged and older population (TLSA). Approximately 17% of the self-reported middle-aged and older populations have diabetes and received diagnosis from physicians. The prevalence of diabetes in TLSA is higher than that reported among the population aged 20–79 years from the IDF Diabetes Atlas of Taiwan in 2021 (9). However, another study showed that the prevalence of diabetes was ~40% among the older adults in 2014 (35). The percentage of diabetes patients (17%) in our study falls within the range of the prevalence in these two previous studies (9, 35), indicating that the prevalence of diabetes among the middle-aged and older populations in our study is reasonable.

Table 1 compares the differences in the severity of perceived poor OHRQoL, the prevalence of OHRQoL, and control variables relative to predisposing, enabling, and health needs between rural and urban diabetic patients. The severity of perceived poor OHRQoL among rural diabetic patients is ~24%, while among urban diabetic patients, it is ~16% (*P* < 0.05). The prevalence of OHRQoL among rural diabetic patients was ~30%, while among urban diabetic patients, it was ~21% (*P* < 0.05). There was no significant difference regarding the predisposing (e.g., age and sex) and health need factors (AD:/IADL). However, compared to urban diabetic patients, rural diabetic patients had a higher rate of not having a high school diploma (86.47% for rural patients vs. 68.77% for urban patients) and were living alone (13.53% for rural patients vs. 7.88% for urban patients).

**Table 1:** Comparison of control variables between rural and urban community-dwelling diabetic patients.

**Study variables**	**Rural (*N* = 133)**	**Non-rural (*N* =698)**	**χ^2^/*t***
**Dependent variables**			
The severity of perceived poor Oral Health-related Quality of Life	32 (24.06%)	109 (15.62%)	5.65^*^
Prevalence of poor Oral Health-related Quality of Life	40 (30.08%)	145 (20.77%)	5.58^*^
**Pre-disposing factors**			
**Age**			0.05
50–64	62 (46.62%)	333 (47.71%)	
65+	71 (53.38%)	365 (52.29%)	
**Sex**			0.15
Male	65 (48.87%)	354 (50.72%)	
Female	68 (51.13%)	344 (49.28%)	
**Marital status**			2.17
Married	83 (62.41%)	481 (68.91%)	
Single, devoice, separate, or widow	50 (37.59%)	217 (31.09%)	
**Enabling factors**			
**Employment status**			3.67
Employed	51 (38.35%)	209 (29.94%)	
Unemployed	82 (61.65%)	489 (70.06%)	
**Education status**			17.21^***^
With a high school diploma or above	18 (13.53%)	218 (31.23%)	
Without a high school diploma	115 (86.47%)	480 (68.77%)	
**Living status**			4.46^*^
Living with someone	115 (86.47%)	643 (92.12%)	
Living alone	18 (13.53%)	55 (7.88%)	
**Family/social support**			0.98
Satisfied	117 (87.97%)	590 (84.65%)	
Unsatisfied	16 (12.03%)	107 (15.35%)	
**Health needs**			
**Activities of daily living or Instrumental activities of daily living limitations**			0.08
None	98 (73.68%)	506 (72.49%)	
At least one difficulty	35 (26.32%)	192 (27.51%)	
Comorbidities^a^	1.83 (1.41)	1.79 (1.41)	0.74
**Unhealthy behaviors related to drinking, smoking or chewing betel**			0.13
None	18 (13.53%)	103 (14.76%)	
At least one unhealthy behavior	115 (86.47%)	595 (85.24%)	

^a^Mean and standard deviation in the parenthesis, with t value for the comparison; ^*^P < 0.05.

Table 2 presents the adjusted differences in the severity of perceived poor OHRQoL and the prevalence of poor OHRQoL between rural and urban patients after controlling for the covariates of the predisposing (e.g., age and sex), enabling (e.g., education and employment status), and health need factors (e.g., comorbidities and living alone). Regarding the severity of poor perceived OHRQoL, rural diabetic patients had a higher likelihood than urban diabetic patients (OR: 1.65, 95% CI: 1.02–2.68). Regarding the prevalence of poor OHRQoL, rural diabetic patients had a higher likelihood than urban diabetic residents; however, the difference was not significant (OR: 1.47, 95%CI: 0.95–2.28).

**Table 2:** Difference in poor Oral Health-related Quality of Life between rural and urban diabetic patients (*N* = 831).

**Study variables**	**Severity of perceived poor OHRQoL, OR (95% CI)**	**Prevalence of poor OHRQoL, OR (95% CI)**
**Key independent variable**		
Rural (ref = Urban)	1.65^*^ (1.02–2.68)	1.47 (0.95–2.28)
**Control variables**		
**Predisposing factors**		
aged 65 and above (ref = aged 50–64)	0.83 (0.53–1.31)	1.19 (0.80–1.78)
Female (ref = male)	0.81 (0.51–1.29)	0.85 (0.56–1.28)
Married (ref = single, devoice, separation, or widow)	0.68 (0.44–1.06)	0.67^*^ (0.45–1.00)
**Enabling factors**		
Having a job (ref=unemployment)	0.76 (0.44–1.30)	1.05 (0.66–1.68)
High school diploma or above (ref = without a high school diploma)	0.60^*^ (0.36–1.00)	0.53^**^ (0.33–0.83)
Living alone (ref = living with someone)	1.94^*^ (1.06–3.57)	1.40 (0.79–2.50)
Satisfied with support from relatives and friends (ref = unsatisfied)	0.51^**^ (0.31–0.83)	0.94 (0.58–1.51)
**Health needs**		
At least one ADL or IADL limitation (ref = No ADL or IADL issue)	3.09^***^ (1.97–4.87)	2.10^***^ (1.40–3.15)
Number of comorbidities	1.06 (0.92–1.21)	1.15^*^ (1.01–1.30)
Health behaviors related to drinking, smoking or chewing betel (ref = No)	1.65 (0.88–3.12)	1.11 (0.66–1.88)

^*^P < 0.05, ^**^P < 0.01, ^***^P < 0.001.

Education and ADL/IADL limitations are most notable among all covariates because they are significantly associated with both poor OHRQoL measures. For example, diabetic patients with a high school diploma or above had a lower likelihood of experiencing the severity of perceived poor OHRQoL (OR: 0.60, 95%CI: 0.36–1.00) and a lower prevalence of poor OHRQoL (OR: 0.53, 95% CI: 0.33–0.83) than their counterparts. However, the difficulty in performing at least one ADL/IADL limitation is significantly associated with the severity of perceived poor OHRQoL (OR: 3.09, 95%CI 1.97–4.87) and the prevalence of poor OHRQoL (OR: 2.10; 95%CI: 1.40–3.15) Furthermore, those living alone were more likely to experience the severity of perceived poor OHRQoL than those without (OR: 1.94, 95%CI: 1.06–3.57). Finally, individuals who were satisfied with support from relatives and friends had a lower likelihood of experiencing the severity of perceived poor OHRQoL than those who were not (OR: 0.51, 95%CI: 0.31–0.83).

## Discussion

In summary, the present study found that rural diabetic patients had a higher severity of perceived poor OHRQoL than urban diabetic patients, with a statistical significance. Rural diabetic patients also had a higher prevalence of poor OHRQoL than urban diabetic patients, although the difference was not significant. Dietary control, regular exercise, and medication adherence form a three-leg medical regimen for diabetes control. Reasonable dietary control requires a healthy oral condition to chew and enjoy various foods while obtaining nutrition and maintaining blood sugar in good condition. With poor perceived OHQRoL, such as meal interruption and discomfort while eating due to teeth and denture problems, patients are likely to experience poor functions for food intake, which would increase the challenges of diabetes management.

Taiwan has implemented a single-payer system under a universal health insurance program since 1995. The program provides comprehensive health care coverage, including inpatient and outpatient western medicine, Chinese medicine, and dental and vision care to the population in Taiwan at a low cost. However, access to dental care for rural residents is still challenging due to the difficulty in recruiting and retaining the healthcare workforce in rural areas in Taiwan. As a result, rural individuals had lower dental utilization and fewer numbers of natural teeth than urban individuals (29, 36), which may explain the findings in the present study.

In addition to geographic factors, other social determinants also affect OHRQoL. Consistent with past findings (35, 36), this analysis shows that social support is critical for chronic disease patients and dental utilization, which would improve the OHRQoL for diabetic patients. In addition, individuals with low health literacy were associated with poor oral health conditions (37). Our findings showed that diabetic patients with a high school diploma and above were less likely to experience poor OHRQoL than those without a high school diploma. Furthermore, evidence showed misconceptions about oral health in the rural population in Taiwan (38). In our study sample, ~86% of diabetic patients in rural areas did not have a high school diploma, and the low education level probably contributes to the misconception. Hence, it is crucial to provide oral health education for rural diabetic patients.

Furthermore, diabetic patients having at least one ADL or IADL limitation were at a higher risk of poor OHQRoL in both measures than those without ADL or IADL limitations. Our findings are consistent with the findings in the literature. Based on Japanese older patients living with family, the study showed that ADL was related to poor OHQRoL (39). Furthermore, a longitudinal study conducted in England, which tracked individuals for two decades, found that the number of natural teeth prevented individuals from losing IADL capacities (40). The above evidence indicated a potential bilateral relationship between poor oral health conditions and ADL or IADL limitations.

## Limitations

The present study has some limitations. First, the rurality was defined by the residential location of individuals who received an interview at the time. Given a cross-sectional study, we could not know the length of time the individuals lived in rural areas. In addition, based on a cross-sectional study design, we were not able to track the change of OHQRoL for individuals from time to time or identify the causal relationship between diabetes and ORQRoL. In addition, diabetic patients included in the study (with OHIP data) were younger and had fewer ADL/IADL limitations than those excluded (without OHIP data). Therefore, the generalizability of the findings in the present study to other populations must be cautious. Finally, the number of dentists or primary care physicians per 1,000 population is likely associated with access to oral health; however, NC_TLSA does not provide residential areas, making merging with other data impossible.

Despite the limitations discussed above, our findings provide implications for policies and future research. The impact of oral health on diabetes control and other chronic conditions was documented decades ago (7, 8). However, policies that address integrating oral health care into primary care practice are largely ignored in Taiwan. In 2001, Taiwan implemented a diabetes pay-for-performance program that financially motivated physicians if their patients received a list of recommended exams, such as an eye exam and hemoglobin A1c. As a result, rural–urban disparities in recommended exams were reduced (41), indicating that healthcare providers responded to payment incentives. However, the diabetes pay-for-performance program does not include dental referrals or assessments as part of quality performance measures. As diabetes control and oral health affect each other (7, 8), providing financial incentives for a dental referral or assessment for diabetes care through the payment system would help diabetic patients receive dental care and further reduce rural-urban disparities in OHRQoL in the future. For example, in 2014, the Department of Health and Human Services Administration in the United States launched interdisciplinary collaboration between dentists and primary care practitioners through meaningful information exchange, referral, and patient/population-centered care (42). The policy facilitated clinics to refer patients to dentists or to include oral health assessment at the primary care clinics, especially those in rural or underserved areas (43). Furthermore, oral health education at the early-stage of childhood, any stage of adulthood, or during follow-up physician appointments is critical to enhancing self-oral health care. Finally, the present study found that rural patients experienced the severity of poor perceived OHRQoL. Future studies that investigate the impact of poor OHRQoL on diabetes control and diabetes-related complications are recommended.

## Conclusions

Oral health and diabetes are both listed as top priorities for improving rural population health (44). Overall, rural diabetic patients had a poor OHQRoL than those in urban areas. Given the bidirectional relationship between oral health and diabetes control, improving oral health in rural areas may serve as a critical avenue to improve the quality of diabetes care in rural areas.

## Data availability statement

The data analyzed in this study is subject to the following licenses/restrictions: The datasets used and analyzed in the present study are not publicly available but are available from the corresponding author on reasonable request with the permission of the Ministry of Health and Welfare, Taiwan. Requests to access these datasets should be directed to chenhf@kmu.edu.tw.

## Ethics statement

The study was approved by the Institutional Review Board at Kaohsiung Medical University Hospital in Taiwan (KMUHIRB- E(I)-20210211).

## Author contributions

HFC conceptualized the study, obtained funding, designed the study, conducted a quality check, wrote the first draft of the manuscript, and revised the manuscript. YTL conducted a literature review, analyzed the data, interpreted the findings, and reviewed and revised the manuscript. JYL conducted a literature review, managed administration for the project, prepared tables, and reviewed and revised the manuscript. HEL conceptualized the study, designed the study, interpreted the findings, supervised the project, and reviewed and revised the manuscript. All authors have read and approved the final version of the manuscript.
